# Selective Separation of Zr(IV) from Simulated High-Level Liquid Waste by Mesoporous Silica

**DOI:** 10.3390/nano14010013

**Published:** 2023-12-20

**Authors:** Yifu Hu, Xue Bai, Yan Chen, Wentao Wang, Qi Chen, Zhi Cao, Taihong Yan

**Affiliations:** Department of Radiochemistry, China Institute of Atomic Energy, Beijing 102413, China

**Keywords:** mesoporous silicas, zirconium, high-level liquid waste

## Abstract

The efficient separation of Zr(IV) ions from strong acidic and radioactive solutions is a significant challenge, especially in the context of the aqueous reprocessing of nuclear fuels. The complexity of such solutions, which are often characterized by high acidity and the presence of radioactive elements, poses formidable challenges for separation processes. Herein, several mesoporous silicas (HMS, MCM-41, KIT-6, and SiO_2_-70 Å) with excellent acid and radiation resistance properties were employed as sorbents to remove Zr(IV) ions from simulated high-level liquid waste. The batch experiments were designed to investigate the influence of adsorption time, HNO_3_ concentration, initial Zr(IV) concentration, adsorbent dosage, and temperature on the adsorption behavior of Zr(IV). The results indicate that the adsorption equilibrium time of mesoporous silica materials was approximately 8 h, and all the adsorption processes followed the pseudo-second-order kinetics equation. The isotherms of Zr(IV) adsorption by KIT-6 exhibited good agreement with the Langmuir model, while the Freundlich model could be utilized to fit the adsorption on HMS, MCM-41, and SiO_2_-70 Å. The adsorption capacity of MCM-41 for Zr(IV) in 3 mol/L HNO_3_ was 54.91 mg/g, which is three times the adsorption capacity reported for commercial silica gel (17.91 mg/g). The thermodynamic parameters indicate that the adsorption processes for zirconium are endothermic reactions. Furthermore, the mesoporous silicas exhibited a pronounced selectivity in the adsorption of Zr(IV) within a simulated high-level liquid waste containing 10 co-existing cations (3 mol/L HNO_3_). This suggests that mesoporous silicas have great potential for Zr(IV) removal in actual radioactive liquids with high acidity during spent fuel reprocessing.

## 1. Introduction

Spent nuclear fuel (SNF) refers to depleted nuclear fuel that has been removed from nuclear reactors and contains a substantial number of radioactive nuclides. One of the primary objectives of nuclear fuel reprocessing is to separate and extract these radioactive nuclides, with the aim of enhancing the management and disposal of radioactive waste. The plutonium uranium redox extraction (PUREX) process is the only commercial technique used in nuclear fuel reprocessing plants worldwide. It is primarily based on the substantial disparity in the extraction capacity of the extractant tributyl phosphate (TBP) for uranium, thorium, neptunium, plutonium, and their fission products in various oxidation states, thus enabling the purification and separation of these elements. The solution resulting from the dissolution of spent nuclear fuel will contain a notable concentration of Zr(IV), given its frequent use as the cladding material for fuel rods. Simultaneously, as a consequence of fission reactions, elements like uranium and plutonium in spent fuel undergo nuclear fission, producing a range of fission products, including isotopes of Zr(IV). Within the PUREX process, Zr(IV) can undergo a reaction with the radiolysis products of TBP and alkane diluents, resulting in the formation of a viscous emulsion [1,2,3] at the interface of the organic and aqueous phases, commonly known as “crud” [4,5]. The formation of crud not only reduces the recovery and purity of uranium and plutonium but also hastens the decomposition of the extractant, thereby hindering the normal production process [6,7]. Furthermore, the PUREX process yields a considerable quantity of Zr(IV)-containing high-level liquid waste (HLLW), and the presence of Zr(IV) in HLLW could initiate the formation of a third phase during solvent extraction. During the solvent extraction treatment of high-level liquid waste (HLLW), the presence of Zr(IV) may lead to the formation of a third phase, the immiscible second organic phase, which arises between the organic phase and the aqueous phase [8,9]. The third phase may cause clogging and corrosion problems in equipment during the liquid–liquid extraction process. Hence, the pre-removal of Zr(IV) emerges as an effective strategy for intercepting the formation pathway of both the crud and the third phase. This ensures a more seamless operation for the PUREX process and facilitates the treatment of high-level liquid waste.

Over the past few decades, researchers have developed diverse adsorbents [10,11], including organic ion exchange resins [12,13,14,15,16], sodium alginate gel [17], silicon-based materials [18,19,20,21,22,23,24,25,26,27,28], zeolites [29], and hydrous ferric oxide [30]. However, most of these adsorbents suffer from poor selectivity, poor acid resistance (only being effective in a narrow pH range in most cases), a low adsorption capacity, long adsorption equilibrium times, and poor radiation resistance, which limits their practical application. As an example, Takashi et al. [17] explored the adsorption behavior of Zr(IV) in alginate polymer gel. The findings indicated that the gel exhibited a stronger affinity for Zr(IV) compared to other fission products and actinides such as Sr, Co, U, Fe, etc. Consequently, 95% of the Zr(IV) could be effectively removed from the spent fuel solutions. In another study, Zhang et al. [20] synthesized a silica gel with a high specific surface area (998 m^2^/g) for the adsorption and separation of Zr(IV) in simulated high-level radioactive liquid waste. The results revealed that the maximal adsorption capacity of the silica gel for Zr(IV) reached as high as 31.4 mg/g. However, it is worth noting that the silica gel exhibited sluggish kinetics, with an adsorption equilibrium time exceeding 50 h. Constructing an absorbent capable of rapidly and effectively separating Zr(IV) from highly acidic and radioactive solutions in spent fuel reprocessing plants remains a formidable challenge.

Silica gel has garnered significant attention among inorganic materials due to its notable selectivity for Zr(IV) in fission products. Silica gel possesses a highly porous structure, with these pores offering a multitude of adsorption sites that facilitate the capture of target substances. Moreover, the pore structure of silica gel can be tailored by adjusting the preparation conditions, providing a versatility that enables selective adsorption of different types of target substances. In addition to its porous structure, silica gel exhibits commendable chemical stability and is compatible with a broad range of acidity levels. However, the adsorption capacity of most silica gels for Zr(IV) is suboptimal due to their limited specific surface area and singular surface structure. Additionally, achieving adsorption equilibrium with these silica gels takes a long time. These shortcomings significantly hinder the practical application of silica gels in spent fuel reprocessing. Therefore, it is necessary to explore silica gels with larger specific surface areas and suitable structures to effectively separate Zr(IV).

In this paper, the adsorption of Zr(IV) in a nitric acid system was studied using a variety of different specifications of mesoporous silicas, such as hierarchically mesoporous silica (HMS), MCM-41, KIT-6, and SiO_2_-70 Å. Batch experiments were adopted to evaluate the adsorption performance of these mesoporous silicas for Zr(IV). In addition, the selectivity of mesoporous silicas for Zr(IV) adsorption in a simulated high-level liquid waste was investigated. The experimental data were subjected to fitting using both a kinetic model and a thermodynamic model to further elucidate the adsorption properties concerning Zr(IV). Finally, ^29^Si magic-angle spinning nuclear magnetic resonance spectra (^29^Si MAS NMR) and X-ray photoelectron spectroscopy (XPS) techniques were used to analyze the interaction mechanism between mesoporous silicas and Zr(IV) during the adsorption process.

## 2. Materials and Methods

### 2.1. Reagents and Materials

All reagents were utilized as received without undergoing additional purification. Hierarchically mesoporous silicas (HMS), MCM-41, KIT-6, and SiO_2_-70 Å (pore size 70 Å), were purchased from Jiangsu XFNANO Nano Materials Tech Co., Ltd. (Jiangsu, China). Zirconium nitrate pentahydrate (Zr(NO_3_)_4_·5H_2_O), sodium nitrate (NaNO_3_), ferric nitrate nonahydrate(Fe(NO_3_)_3_·9H_2_O), silver nitrate (AgNO_3_), strontium nitrate (Sr(NO_3_)_2_), caesium nitrate (CsNO_3_), barium nitrate (Ba(NO_3_)_2_), lanthanum nitrate hexahydrate (La(NO_3_)_3_·6H_2_O), cerium nitrate hexahydrate (Ce(NO_3_)_3_·6H_2_O), neodymium nitrate hexahydrate (Nd(NO_3_)_3_·6H_2_O), samarium nitrate hexahydrate (Sm(NO_3_)_3_·6H_2_O), and europium nitrate hexahydrate (Eu(NO_3_)_3_·6H_2_O) were purchased from Macklin. HNO_3_ (68 wt%) was purchased from Sinopharm Chemical Reagent Co., Ltd (Beijing, China). with the grade of A.R.

### 2.2. Characterization

The Brunauer–Emmett–Teller (BET) surface area of the used silica materials was determined using a Surface Area and Porosity Analyzer (ASAP 2460). The morphologies of the samples were observed using scanning electron microscopy (SEM) with a Hitachi S4800 instrument operating at 10 kV. Fourier transform infrared (FTIR) measurements were carried out in the range of 4000–400 cm^−1^ on a Thermo Nicolet 6700 spectrometer. X-ray photoelectron spectroscopy (XPS) spectra were obtained using a Thermo Scientific Escalab 250Xi electron spectrometer. The binding energies were referenced from the carbon C1s line at 284.8 eV originating from adventitious carbon. ^29^Si MAS NMR spectroscopy was conducted using a Bruker Ascend III 400 WB. The bath shaker (SHA-B) was procured from Hangzhou Qinlai Experimental Instrument Co., Ltd (Hangzhou, China).

### 2.3. Batch Experiments

In this study, a 100 mg/L Zr(IV) in 3 mol/L HNO_3_ solution was prepared for batch experiments. A specific quantity of mesoporous silica was weighed and combined with the Zr(IV)-containing solution in glass bottles. The bottles were then securely positioned in a bath shaker and agitated at 200 rpm at a designated temperature. Following the adsorption of Zr(IV) by mesoporous silica, the solutions were subsequently separated using a filter with a pore size of 0.22 μm. The filtrate was measured using ICP-OES (Thermo Scientific ICAP7400 Duo, Waltham, MA, USA). Under the same experimental conditions, three parallel samples were conducted.

The adsorption selectivity of the adsorbent was also investigated in simulated high-level liquid waste (HLLW) in the presence of other competing ions. The concentrations of metal ions, including Ag^+^, Ba^2+^, Cs^+^, Ce^3+^, Eu^3+^, Fe^3+^, La^3+^, Nd^3+^, Sm^3+^, Sr^2+^, and Zr^4+^ in the 3 mol/L HNO_3_ phase, were determined.

A designated amount of mesoporous silica was blended with the required volume of Zr(NO_3_)_4_ aqueous solution within a 10 mL glass bottle. Following this, the mixture was shaken in a water bath shaker at the prescribed temperature. The mesoporous silica was isolated using a 0.22 μm filter membrane for subsequent characterization. The filtrate was analyzed for residual Zr(IV) content using ICP-OES (Thermo Scientific ICAP7400 Duo).

The equilibrium adsorption capacity *Q*_e_ (mg/g), adsorption efficiency *RE* (%), and distribution coefficient *K*_d_ (mL/g) were calculated according to the follow equations:

(1) $$Q_{e}=\frac{V}{m}\times (C_{0}-C_{e})$$

(2) $$K_{d}=\frac{V}{m}\times \frac{\left(C_{0}-C_{e}\right)}{C_{e}}$$

(3) $$RE=\frac{\left(C_{0}-C_{e}\right)}{C_{0}}\times 100\%$$

where *C*_0_ and *C*_e_ are initial and equilibrium concentrations of Zr(IV) in aqueous solution (mg/L); *m* is the mass of mesoporous silica (g); and *V* is the volume of aqueous solution (L).

## 3. Results and Discussion

### 3.1. Characterization

**Figure 1:**
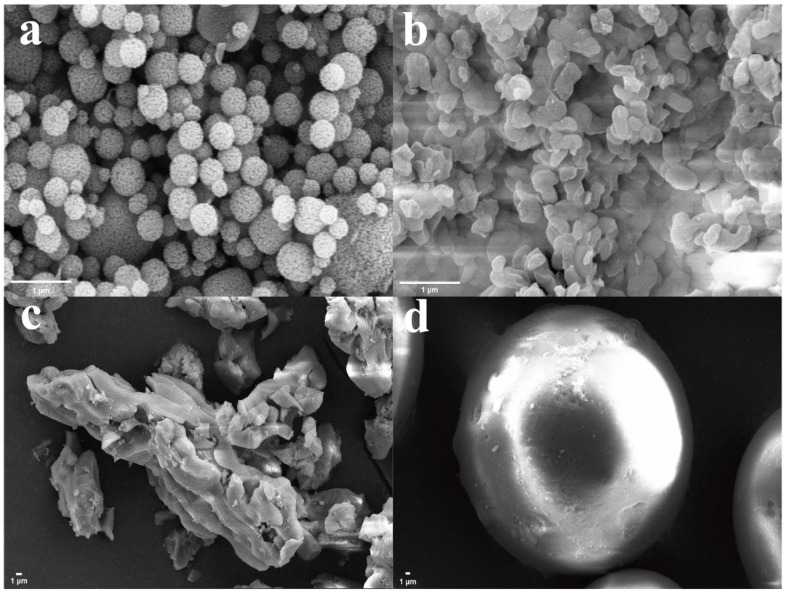
SEM images of (**a**) HMS, (**b**) MCM-41, (**c**) KIT-6, and (**d**) SiO_2_-70 Å.

Figure 1 illustrates scanning electron microscopy (SEM) images depicting the characteristics of mesoporous silicas. Within these images, the surface of HMS exhibits discernible voids and cavities. Conversely, MCM-41 shows an irregular, crystallized two-dimensional flake morphology. The KIT-6 sample displays a disorderly arrangement of layers and aggregates, contributing to its unique structural features. In contrast, SiO_2_-70 Å presents as spherical particles with diameters ranging in the order of hundreds of micrometers.

**Figure 2:**
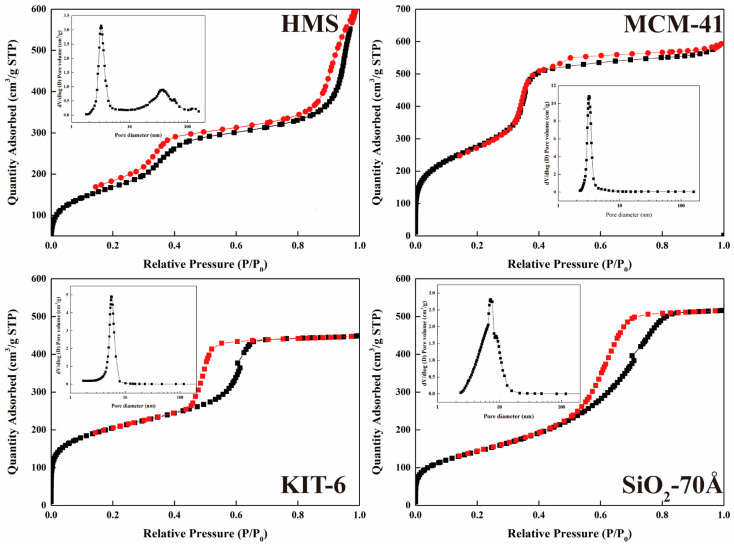
N_2_ adsorption–desorption isotherm of HMS, MCM-41, KIT-6, and SiO_2_-70 Å.

The BET analysis was performed to determine the pore size distribution and specific surface area of the mesoporous silica. The results obtained from both the N_2_ adsorption–desorption isotherms and the corresponding pore size distribution curves (Figure 2) reveal distinctive characteristics for each material. In the case of MCM-41, KIT-6, and SiO_2_-70 Å, the N_2_ adsorption–desorption isotherms exhibit a type IV curve marked by a significant hysteresis loop, suggesting a mesoporous structure. This observation aligns with the mesoporous nature expected for these materials. Concurrently, the pore size distribution curves further confirm the presence of mesopores in these samples. Contrastingly, the analysis results of HMS reveal a more intricate structural composition. While the N_2_ adsorption–desorption isotherm still displays a type IV curve with a significant hysteresis loop, suggesting a mesoporous structure, the pore size distribution curve indicates the combined presence of mesopores and macropores. This distinction in pore size distribution implies a more complex arrangement within the HMS material, encompassing both mesoporous and macroporous characteristics (Table 1).

**Figure 3:**
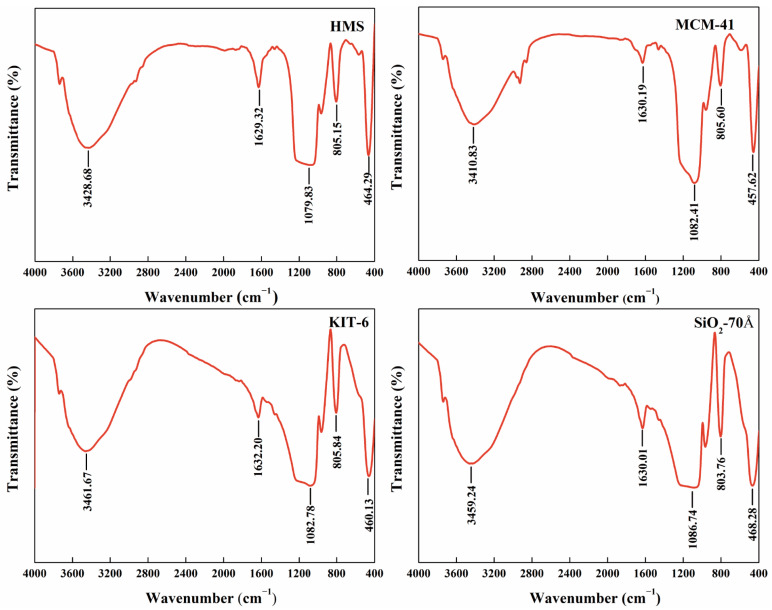
Infrared spectra of mesoporous silica.

Figure 3 displays the infrared spectrum of the samples. The infrared spectrum reveals that the characteristic absorption peaks of the four samples were located at the same position. The strong absorbance at ~1080 cm^–1^ is attributed to the anti-symmetric stretching vibration of the Si-O-Si groups, while the absorption peak at ~805 cm^–1^ corresponds to the symmetric stretching vibration of the silico-oxygen tetrahedron. The absorption peak at ~460 cm^–1^ is attributed to the stretching vibration of the Si-O-Si groups [31]. A broad peak ranging from ~3410 cm^–1^ to ~3460 cm^–1^ is attributed to the presence of surface hydroxyl groups [32].

### 3.2. Kinetics

**Figure 4:**
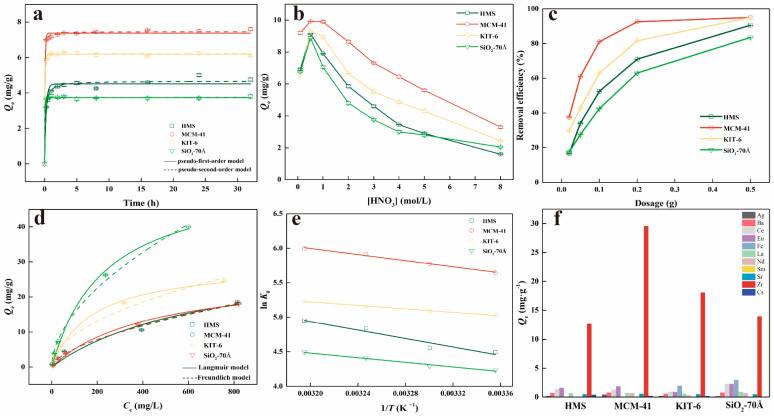
(**a**) Effect of the contact time on the adsorption of Zr(IV). (**b**) Effect of HNO_3_ concentration on the adsorption of Zr(IV). (**c**) Effect of the dosage of the adsorbents on the adsorption of Zr(IV). (**d**) Experimental isotherms for the adsorption of Zr(IV) at 298 K fitted by Langmuir and Freundlich models. (**e**) Effect of the temperature on the adsorption of Zr(IV). (**f**) Adsorption capacity of different element in the simulated high-level liquid waste.

The effect of contact time on the adsorption performance of the silica adsorbents for Zr(IV) is exhibited in Figure 4a. It can be seen that all four mesoporous silicas basically reached the adsorption equilibrium at approximately 8 h. Given these results, subsequent experiments were carried out using silica adsorbents with an adsorption time of 8 h to ensure the attainment of maximum Zr(IV) adsorption.

To investigate the adsorption kinetics of the silica adsorbents for Zr(IV), both the pseudo-first-order kinetic and pseudo-second-order kinetic were fitted. The pseudo-first-order model and pseudo-second-order model formulas are as follows [33,34]:

(4) $$Q_{t}=Q_{e}(1-e^{{-k}_{1}t})$$

(5) $$Q_{t}=\frac{{kQ}_{e}^{2}t}{1+kQ_{e}t}$$

where *Q_e_* is the equilibrium adsorption capacity of the adsorbents (mg/g), *Q_t_* is the adsorption capacity of the adsorbents at time *t* (mg/g), *t* is the adsorption time (h), and *k*_1_ (min^−1^) and *k*_2_ (mg·g^−1^·min^−1^) are the pseudo-first-order kinetic constant and pseudo-second-order kinetic constant, respectively.

From the curve in Figure 4a, the values of *k*_1_, *k*_2_, and the corresponding *Q_e_* can be directly determined through fitting. Table 2 provides a summary of these fitting parameters. As demonstrated in Table 2, the Zr(IV) adsorption on mesoporous silicas was accurately described by the pseudo-second-order kinetic model (*R*^2^ = 0.99), indicating the dominance of a chemisorption process.

### 3.3. Effects of Nitric Acid Acidity

Figure 4b shows the effect of HNO_3_ concentration on the adsorption capacity of Zr(IV). The adsorption capacity of Zr(IV) by mesoporous silicas showed an initial increase followed by a decrease with an increasing concentration of HNO_3_ in the solution from 0.1 mol/L to 8 mol/L, as depicted in Figure 4b. An optimal HNO_3_ concentration of 0.5 mol/L was identified for achieving maximum adsorption capacity. The observed parabolic trend in adsorption capacity can be attributed to the hydrolysis and polymerization behavior of Zr(IV) in the aqueous solution. Previous studies have reported that Zr(IV) undergoes hydrolysis to form monomeric species, including Zr(OH)^3+^, Zr(OH)_2_^2+^, Zr(OH)_3_^+^, and Zr(OH)_4_(aq), as well as a range of polymeric species, such as Zr_2_(OH)_6_^2+^, Zr_3_(OH)_4_^8+^, Zr_3_(OH)_5_^7+^, and Zr_4_(OH)_8_^8+^ [35,36,37,38]. The specific species formed depend on the acidity of the solution [36,37] and the concentration of Zr(IV) [39,40]. When the HNO_3_ concentration was 0.1 mol/L, Zr(IV) predominantly existed in the form of tetramers, with only a small proportion of monomers and polynuclear species [36]. The larger size of the tetra-/polynuclear species may hinder their adsorption on the surface of mesoporous silicas due to the limited availability of adsorption sites [41]. At an HNO_3_ concentration of 0.5 mol/L, there was an increase in the content of monomers, and the competitive adsorption of H^+^ appeared to be relatively low. Consequently, the equilibrium adsorption capacity (*Q*_e_) reached its maximum values under these conditions. When the HNO_3_ concentration further increased, the competitive adsorption of H^+^ intensified, leading to a decrease in the adsorption capacity of Zr(IV) on the adsorbent. This phenomenon is similar to that seen in the results observed by Lin et al. [22].

### 3.4. Effects of Adsorbent Dosage

To investigate the impact of adsorbent dosages on the efficiency of Zr(IV) adsorption, a series of adsorption experiments were conducted by incrementally increasing the dosage of adsorbents while maintaining a fixed Zr(IV)-containing solution volume of 10 mL ([HNO_3_] = 3 mol/L, [Zr] = 100 mg/L). Figure 4c illustrates the influence of adsorbent dosage on the adsorption capacity for Zr(IV). Following an 8 h contact period at 25 °C, dosages of 0.2 g and 0.5 g of MCM-41 led to Zr(IV) removal efficiencies of 92.5% and 95.1%, respectively.

### 3.5. Isotherm

The adsorption isotherms of mesoporous silicas on Zr(IV) were examined by progressively elevating the initial Zr(IV) concentration while maintaining a sorbent/liquid ratio of 1:100. The concentration of Zr(IV) at adsorption equilibrium was measured in a 3 mol/L HNO_3_ solution at 298 K. The results are illustrated in Figure 4d. The Langmuir adsorption model and Freundlich adsorption model [42,43] were employed to analyze the adsorption mechanism of Zr(IV) on the silica-based materials.

The Langmuir model and the Freundlich model are as follows:

(6) $$Q_{e}=\frac{Q_{m}K_{L}C_{e}}{1+K_{L}C_{e}}$$

(7) $$Q_{e}=K_{F}{C_{e}}^{\frac{1}{n}}$$

In the equation, *C_e_* (mg/L) is the equilibrium concentration and *Q_e_* (mg/g) is the equilibrium adsorption capacity of adsorption, respectively; *Q_m_* (mg/g) represents the maximum adsorption capacity of monolayer adsorption in the Langmuir model; *K_L_* (L/mg) represents the Langmuir adsorption constant; and *K_F_* [(mg^1−n^·L^n^)/g] and *n* represent the Freundlich adsorption constant.

A comparison of the fitting results from the Langmuir and Freundlich isotherm models revealed that the adsorption process of KIT-6 on Zr(IV) exhibited a better fit with the Langmuir model. The adsorption of HMS, MCM-41, and SiO_2_-70 Å can be effectively described by the Freundlich model, exhibiting excellent fits with coefficients of determination (*R*^2^) greater than 0.98 for each adsorbent. The adsorption capacity of MCM-41 for Zr(IV) in 3 mol/L HNO_3_ was 54.91 mg/g, which is three times the adsorption capacity reported for commercial silica gel (17.91 mg/g) [21]. The parameter *n* in Table 3, which represents the favorability and intensity of the adsorption, demonstrates a favorable adsorption when *n* > 1.

In order to investigate the thermodynamics of the adsorption process, the adsorption thermodynamic parameters at each temperature were calculated using the Van’t Hoff equation as follows [44]:

(8) $$∆G^{0}=-RTlnK_{0}$$

The Gibbs free energy change (∆*G*^0^) of the adsorption can be calculated by the following formula:

(9) $$∆G^{0}=∆H^{0}-T∆S^{0}$$

(10) $$lnK_{0}=-\frac{{∆H}^{0}}{RT}+\frac{{∆S}^{0}}{R}$$

∆*H*^0^ represents the enthalpy change (kJ/mol), ∆*S*^0^ denotes the entropy change (J·mol^–1^·K^–1^) associated with the adsorption process, *K*_0_ is the thermodynamic equilibrium constant, R stands for the gas constant (8.314 J·mol^–1^·K^–1^), and T signifies the thermodynamic temperature (K).

Values of *K*_0_ at different temperatures could be obtained by fitting a plot of ln(*K_d_*) versus Ce at various temperatures (Figure S1). When *C_e_* is infinitely close to 0, the intercept can be regarded as ln(*K*_0_) [44].

It can be seen from Table 4 that all the values of ∆*H*^0^ are positive, indicating that the reaction is endothermic. All the Gibbs free energies (∆*G*^0^) < 0, suggesting that the reaction can occur spontaneously.

### 3.6. Adsorption Selectivity

The effect of coexisting competing ions on Zr(IV) adsorption by mesoporous silicas in 3 mol/L HNO_3_ solutions is shown in Figure 4f. The composition of cations is provided in Table S1. Figure 4f demonstrates the remarkable selectivity of mesoporous silicas for Zr(IV), as well as their significant adsorption capacity for transition-metal ions. The adsorption capacity of the mesoporous silicas for competitive ions in the solution is strongly influenced by their ionic potential, which is determined by the charge-to-radius ratio. The higher the ionic potential of an ion, the more readily it can be absorbed. This explains the exceptional selectivity of the employed mesoporous silicas for Zr(IV). The excellent selectivity of mesoporous silicas for Zr(IV) in highly acidic conditions renders them highly suitable for the selective removal of Zr(IV) from real high-level radioactive liquid waste.

### 3.7. Mechanism

**Figure 5:**
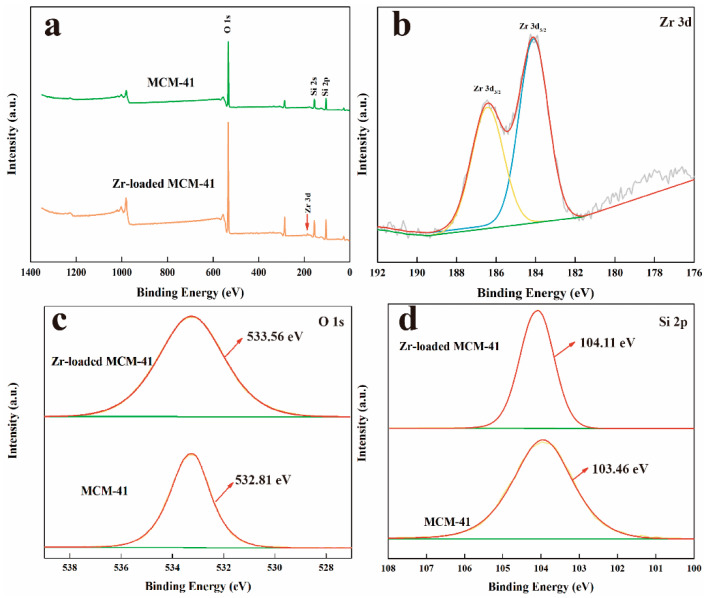
(**a**) XPS spectra of MCM-41 and Zr-loaded MCM-41, (**b**) XPS spectra of Zr 3d, (**c**) O 1s, and (**d**) Si 2p.

The XPS technique was employed to analyze the interaction mechanism of the adsorption process. Figure 5 reveals the presence of the primary constituent elements, including Si and O, in MCM-41. The survey spectra of MCM-41 before and after adsorption, along with signals of Zr 3d, Si 2p, and O 1s, were recorded. The Zr(3d) core level characteristic peak after adsorption could be deconvoluted into two Gaussian peaks: 186.4 eV for Zr(3d_3/2_) and 184.07 eV for Zr(3d_5/2_) (inset of Figure 5b). A slight shift in Si 2p and O 1s XPS spectra was observed over MCM-41 before and after Zr(IV) adsorption, which is possibly attributable to the strong affinities between Zr(IV) ions and the mesoporous silica framework, consistent with the literature [45,46,47].

**Figure 6:**
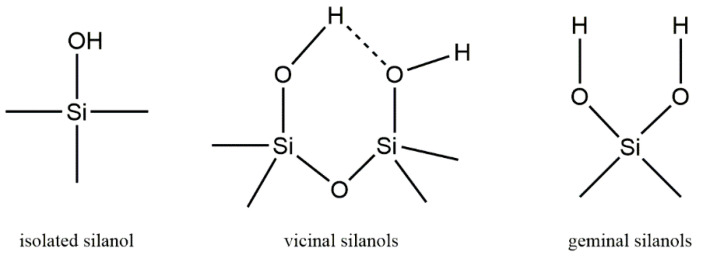
The types of groups on the surface of silica gel.

The surface of silica consists of diverse hydroxyl groups, which can be categorized as isolated and geminal silanols [48]. Geminal silanols consist of two hydroxyl groups attached to a single silicon atom, while isolated silanols have only one hydroxyl per silicon atom and may form hydrogen bridges (vicinal silanols) depending on the distance between silanols and the local geometric structure (Figure 6). In theory, the adsorption capacity of Zr(IV) will also change with the difference in silicon hydroxyl group, so it is necessary to study the types of silicon hydroxyl groups on the surface of silica adsorbents. The utilization of solid-state ^29^Si NMR to identify surface silicon hydroxyl groups within different silica gels was first reported by Maciel et al. [49,50,51]. The ^29^Si NMR spectrum revealed isolated silanols at approximately 100 ppm and geminal silanols at around 91 ppm.

The resonance areas in each ^29^Si NMR spectrum were obtained through a curve fitting routine, and then deconvolution was performed (Figure S2). The relative isolated silanol area (%I) is the resonance area of ~100 ppm (I) divided by the sum of I and the ~91 ppm geminal silanol resonance areas (G) (Table 5). The percentage of isolated silanols per total silanols present is estimated from these areas as well, where the isolated silanol area (% I OH) = I/(2 × G + I) [52]. The number of isolated silanols on the surface is important, because they lead to high activity [53]. Therefore, the variation in the content of isolated silanols among mesoporous silicas may be responsible for the differences in their adsorption capacity for Zr(IV).

## 4. Conclusions

The ability of mesoporous silicas to adsorb Zr(IV) from aqueous solutions was investigated. All the adsorbents achieved adsorption equilibrium within 8 h and conformed well to the pseudo-second-order kinetic model. The isotherms of Zr(IV) adsorption by KIT-6 were well fitted with the Langmuir model, while the other absorbents exhibited a good fit with the Freundlich model. At a temperature of 298 K and a fixed sorbent/liquid ratio of 1:100, the calculated maximum adsorption capacities for Zr(IV) in a 3 mol/L HNO_3_ solution were 32.01 mg/g for HMS, 54.97 mg/g for MCM-41, 29.77 mg/g for KIT-6, and 27.28 mg/g for SiO_2_-70 Å, respectively. The thermodynamics of Zr(IV) adsorption on silica-based materials was a spontaneous endothermic process. The results of the ^29^Si NMR spectrum indicate that the variation in the content of isolated silanols among mesoporous silicas may be responsible for the differences in their adsorption capacity for Zr(IV). In addition, the adsorbents showed highly selective adsorption of Zr(IV) in a simulated high-level liquid waste containing 10 co-existing cations (Ag^+^, Ba^2+^, Cs^+^, Ce^3+^, Eu^3+^, Fe^3+^, La^3+^, Nd^3+^, Sm^3+^, Sr^2+^) in 3 mol/L HNO_3_ solution. This suggests that mesoporous silicas have significant potential for the selective separation of Zr(IV) in actual high-level liquid waste with high acidity during spent fuel reprocessing.

## Figures and Tables

**Table 1:** Parameters of several mesoporous silicas.

Name	BET Specific Surface Area (m²/g)	Average Pore Size (Å)
HMS	612.21	59.71
MCM-41	1014.69	36.16
KIT-6	733.30	37.88
SiO_2_-70 Å	518.06	61.60

**Table 2:** Kinetic parameters of mesoporous silicas for the adsorption of Zr(IV).

Name of Material	Pseudo-First-Order Model				Pseudo-Second-Order Model			
	*Q*_e_ (mg/g)	*k*_1_ (min^−1^)	*R* ^2^	*χ^2^*	*Q_e_* (mg/g)	*k*_2_ (min^−1^)	*R* ^2^	*χ^2^*
HMS	4.504	4.204	0.960	7.64 × 10^−2^	4.666	1.611	0.984	3.08 × 10^−2^
MCM-41	7.379	11.533	0.994	2.81 × 10^−2^	7.457	6.863	0.998	1.07 × 10^−2^
KIT-6	6.186	12.133	0.998	6.28 × 10^−3^	6.215	18.097	0.998	6.20 × 10^−3^
SiO_2_-70 Å	3.729	15.155	0.996	5.57 × 10^−3^	3.741	34.794	0.996	5.21 × 10^−3^

**Table 3:** Fitting parameters of isotherm for the adsorption of Zr(IV) by several mesoporous silicas.

Adsorbents	Temperature (*K*)	Langmuir Isotherm				Freundlich Isotherm			
		*Q*_m_ (mg/g)	*K*_L_	*R^2^*	*χ^2^*	*K*_F_	*1/n*	*R^2^*	*χ^2^*
HMS	298 K	32.78	1.47 × 10^−3^	0.945	2.94	0.337	0.593	0.985	0.811
MCM-41	298 K	54.91	4.24 × 10^−3^	0.993	2.06	1.273	0.542	0.994	1.644
KIT-6	298 K	29.84	5.54 × 10^−3^	0.996	0.396	0.914	0.502	0.977	2.513
SiO_2_-70 Å	298 K	27.24	2.28 × 10^−3^	0.995	0.258	0.326	0.600	0.996	0.243

**Table 4:** Thermodynamic parameters of Zr onto several mesoporous silicas.

Name of Material	Temperature	∆*H*^0^ (kJ/mol)	∆*S*^0^ (J/mol·K)	∆*G*^0^ (kJ/mol)
HMS	298 K	25.533	147.697	−18.481
	303 K			−19.219
	308 K			−19.958
	313 K			−20.696
MCM-41	298 K	18.433	108.849	−14.004
	303 K			−14.548
	308 K			−15.092
	313 K			−15.637
KIT-6	298 K	10.457	76.878	−12.453
	303 K			−12.837
	308 K			−13.221
	313 K			−13.606
SiO2-70 Å	298 K	13.856	81.588	−10.457
	303 K			−10.865
	308 K			−11.273
	313 K			−11.681

**Table 5:** Relative isolated silanol areas for mesoporous silicas obtained by ^29^Si NMR spectroscopy.

	Isolated Silanol Areas		^29^Si NMR Resonance Chemical Shift	
	(%I)	(%I OH)	Isolated Silanols (ppm)	Geminal Silanols (ppm)
HMS	90.31%	82.33%	−100.43	−90.86
MCM-41	95.72%	91.79%	−100.41	−90.4
KIT-6	95.64%	91.65%	−101.58	−92.13
SiO_2_-70 Å	87.45%	77.69%	−100.71	−91.03

## Data Availability

No additional data are available.

## Supplementary Materials

The following supporting information can be downloaded at: https://www.mdpi.com/article/10.3390/nano14010013/s1. Figure S1: Plots of ln(*K*_d_) versus Ce on silica-based adsorbents at 298K, 303K, 313K, and 323K (adsorbent dosage: 10 g/L, [HNO3]: 3 mol/L, time = 180 min); Figure S2: ^29^Si NMR spectra of (a) HMS, (b) MCM-41, (c) KIT-6, and (d) SiO_2_-70Å; Table S1: component of simulated HLLW.
