# Supplementary material for: Selective Separation of Zr(IV) from Simulated High-Level Liquid Waste by Mesoporous Silica
Source: Nanomaterials (Basel). 2023 Dec 20;14(1):13. doi: 10.3390/nano14010013 (PMC10780328; doi:10.3390/nano14010013)
Supplement: Supplementary file 1 [file nanomaterials-14-00013-s001.zip › nanomaterials-2751508-supplementary.pdf]

nanomaterials

Supplementary Materials

Selective Separation of Zr(IV) from Simulated High-Level Liquid Waste by Mesoporous Silica

Yifu Hu, Xue Bai, Yan Chen, Wentao Wang *, Qi Chen, Zhi Cao and Taihong Yan *

Department of Radiochemistry, China Institute of Atomic Energy, Beijing 102413, China
* Correspondence: wangwt@ciae.ac.cn (W.W.); yanth@ciae.ac.cn (T.Y.)

Table S1. Component of simulated HLLW.

The name of the element	Ag	Ba	Ce	Eu	Fe	La	Nd	Sm	Sr	Zr	Cs
Concentration g/L	0.017	0.3	0.5	0.5	1.9	0.2	0.67	0.14	0.13	0.61	0.4

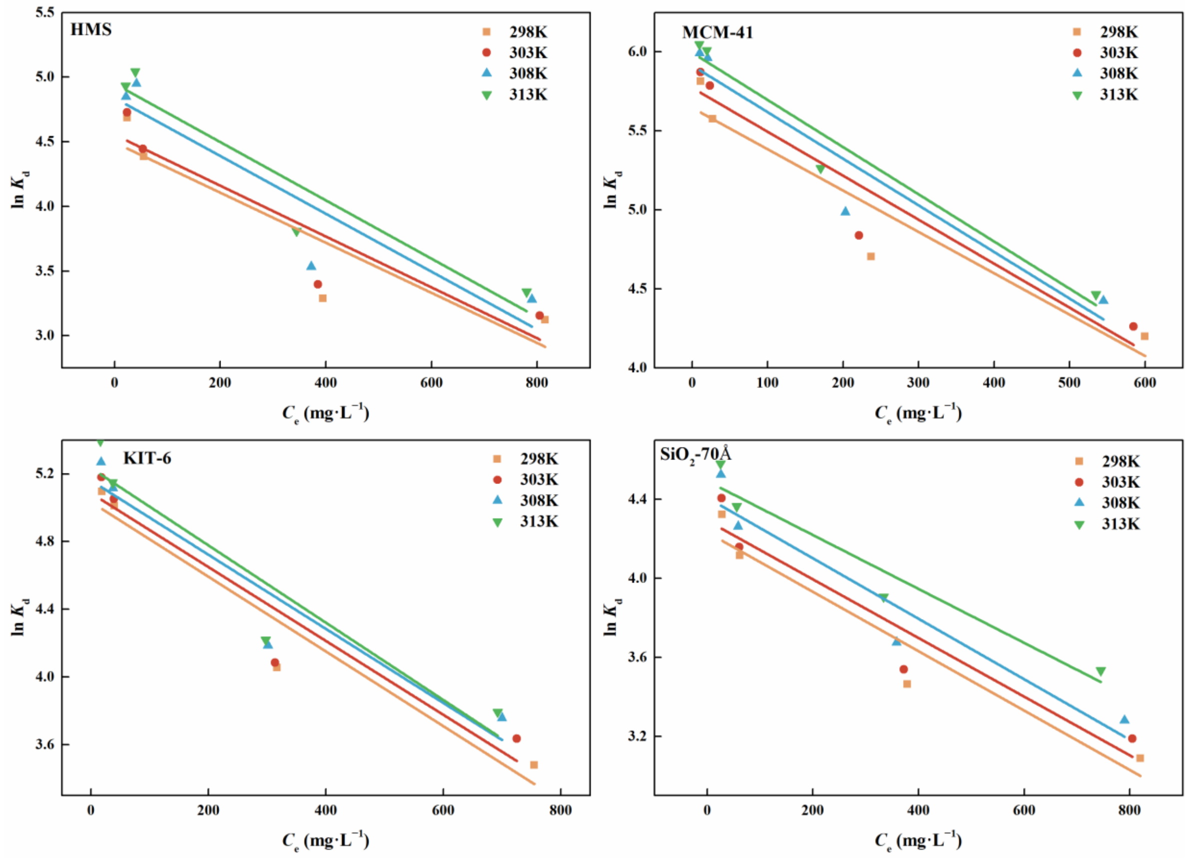

Figure S1. Plots of ln(K_d) versus C_e on silica-based adsorbents at 298K, 303K, 313K, 323K (Adsorbent dosage: 10 g/L, [HNO_3]: 3 mol/L, time=180 min).

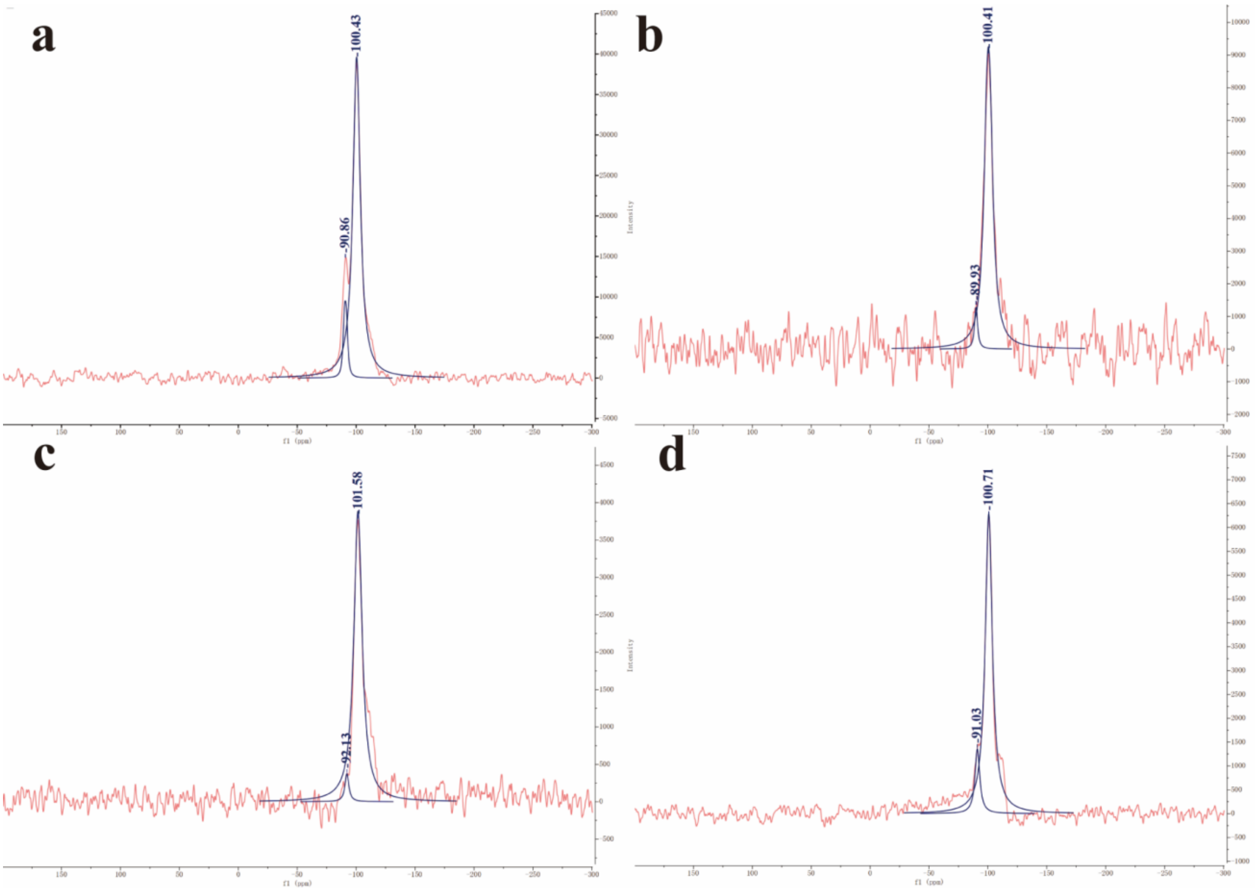

Figure S2. ^{29}Si NMR spectra of (a) HMS, (b) MCM-41, (c) KIT-6, and (d) SiO_2-70Å.
